# Bacteria and Archaea diversity within the hot springs of Lake Magadi and Little Magadi in Kenya

**DOI:** 10.1186/s12866-016-0748-x

**Published:** 2016-07-07

**Authors:** Anne Kelly Kambura, Romano Kachiuru Mwirichia, Remmy Wekesa Kasili, Edward Nderitu Karanja, Huxley Mae Makonde, Hamadi Iddi Boga

**Affiliations:** Institute for Biotechnology Research, Jomo Kenyatta University of Agriculture and Technology, P.O. Box 62000-00200, Nairobi, Kenya; Embu University College, P. O. Box 6-60100, Embu, Kenya; International Centre of Insect Physiology and Ecology (ICIPE), P. O. Box 30772-00100, Nairobi, Kenya; Pure and Applied Sciences, Technical University of Mombasa, P. O. Box 90420-80100, GPO, Mombasa, Kenya; Taita Taveta University College, P.O. Box 635-80300, Voi, Kenya

**Keywords:** Lake Magadi, Hot springs, 16S rDNA, 16S rRNA cDNA, Microbial diversity

## Abstract

**Background:**

Lake Magadi and little Magadi are hypersaline, alkaline lakes situated in the southern part of Kenyan Rift Valley. Solutes are supplied mainly by a series of alkaline hot springs with temperatures as high as 86 °C. Previous culture-dependent and culture-independent studies have revealed diverse groups of microorganisms thriving under these conditions. Previous culture independent studies were based on the analysis of 16S rDNA but were done on less saline lakes. For the first time, this study combined illumina sequencing and analysis of amplicons of both total community rDNA and 16S rRNA cDNA to determine the diversity and community structure of bacteria and archaea within 3 hot springs of L. Magadi and little Magadi.

**Methods:**

Water, wet sediments and microbial mats were collected from springs in the main lake at a temperature of 45.1 °C and from Little Magadi *“Nasikie eng’ida”* (temperature of 81 °C and 83.6 °C). Total community DNA and RNA were extracted from samples using phenol-chloroform and Trizol RNA extraction protocols respectively. The 16S rRNA gene variable region (V4 – V7) of the extracted DNA and RNA were amplified and library construction performed following Illumina sequencing protocol. Sequences were analyzed done using QIIME while calculation of Bray-Curtis dissimilarities between datasets, hierarchical clustering, Non Metric Dimensional Scaling (NMDS) redundancy analysis (RDA) and diversity indices were carried out using the R programming language and the Vegan package.

**Results:**

Three thousand four hundred twenty-six and one thousand nine hundred thirteen OTUs were recovered from 16S rDNA and 16S rRNA cDNA respectively. Uncultured diversity accounted for 89.35 % 16S rDNA and 87.61 % 16S rRNA cDNA reads. The most abundant phyla in both the 16S rDNA and 16S rRNA cDNA datasets included: *Proteobacteria* (8.33–50 %), *Firmicutes* 3.52–28.92 %, *Bacteroidetes* (3.45–26.44 %), *Actinobacteria* (0.98–28.57 %) and *Euryarchaeota* (3.55–34.48 %) in all samples. NMDS analyses of taxonomic composition clustered the taxa into three groups according to sample types (i.e. wet sediments, mats and water samples) with evident overlap of clusters between wet sediments and microbial mats from the three sample types in both DNA and cDNA datasets. The hot spring (45.1 °C) contained less diverse populations compared to those in Little Magadi (81–83 °C).

**Conclusion:**

There were significant differences in microbial community structure at 95 % level of confidence for both total diversity (*P* value, 0.009) based on 16S rDNA analysis and active microbial diversity (*P* value, 0.01) based on 16S rRNA cDNA analysis, within the three hot springs. Differences in microbial composition and structure were observed as a function of sample type and temperature, with wet sediments harboring the highest diversity.

**Electronic supplementary material:**

The online version of this article (doi:10.1186/s12866-016-0748-x) contains supplementary material, which is available to authorized users.

## Background

Extreme environment refers to any setting that exhibits life conditions detrimental to living organisms with respect to its physicochemical properties such as pH, temperature, pressure, nutrient and saline concentration [1]. Extreme physicochemical parameters include acidity (pH <5), alkalinity (pH >9), hyper salinity (salinity >35 %), pressure (>0.1 MPa), high temperature (>40 °C), low temperature (<5 °C), water stress (aw <0.80), and high-radiation environments [2]. The extreme environments are inhabited by organisms referred to as extremophiles that are so well-adapted that they readily grow and multiply [3]. In Kenya, the haloalkaline soda lakes are characterized by exceptionally rich productivity rates presumably because of the high ambient temperatures, high light intensities, availability of phosphates and unlimited access to CO_2_ in these carbonate rich waters [4, 5]. Salinity levels can be as high as 30 % to saturation in Lake Magadi, whereas the pH ranges between 9 and 11.5 [6]. In Lake Magadi, solutes are supplied mainly by a series of alkaline springs with temperatures varying from 33 °C to 86 °C [6, 7].

Previous culture dependent and culture independent studies on Lake Magadi have revealed a dense and diverse population of aerobic, organotropic, halophilic, alkaliphilic, and haloalkaliphilic and alkalitolerant representatives of major bacterial phyla [8–15]. Although conventional microbial cultivation methods have helped shape understanding of physiology and metabolic functions of diverse organisms, they are laborious, time consuming, selective and biased for specific microbial growth. On the other hand, culture - independent studies done on soda lakes in Kenya have been based on the analysis of clone libraries of PCR amplified rDNA. This may not represent an accurate picture of prokaryotic diversity within a given community due to low speed and coverage of a cloning and Sanger-sequencing based approach, which gives a lower number of amplicon sequences compared to the millions of generated by High Throughput Sequencing technologies such as Illumina Sequencing [16]. This is the first culture independent study of the microbial community within the hot springs located around the hypersaline Lakes Magadi and Little Magadi. This study employed Illumina Sequencing of PCR products of both 16S rDNA and 16S rRNA cDNA to obtain a less biased estimation of microbial community within the hot springs’ ecosystem. The main objective of this study was to analyze the targeted total community rDNA and cDNA generated from rRNA so as to compare the total versus active microbial communities within the hot springs of Lake Magadi and Little Magadi in Kenya.

## Methods

### Research authorization

Research authorization was obtained from National Commission for Science, Technology and Innovation (NACOSTI) on 30th August 2013 in Kenya, and permission to conduct research in Lake Magadi was obtained from Kenya Wildlife Services (KWS) on 24th September 2013.

### Study site

Lake Magadi is a hypersaline lake that lies in a naturally formed closed lake basin within the Southern part of the Kenyan Rift Valley. It is approximately 2° 00′ 0″ S and 36° 00′ 0″ E of the Equator at an elevation of about 600 m above sea level [17]. The solutes are supplied mainly by a series of alkaline springs with temperatures as high as 86 °C which are located around the perimeter of the lake. Samples analyzed in this study were collected from three hot springs: one hot spring within the main L. Magadi (02° 00′ 3.7″S 36° 14′ 32″ E) at 45.1 °C and pH 9.8; and two hot springs within Little Magadi “*Nasikie eng’ida”* (01° 43′ 28″S 36° 16′ 21″E), and (01° 43′ 56″ S 36° 17′ 11″ E) at elevations of 611 and 616 m, temperatures of 81 and 83.6 °C and pH range of 9.2 and 9.4 respectively (Table 1).

**Table 1:** Parameters of sampling sites analyzed in this study

Parameter	Latitude °S	Longitude °E	Elevation (m)	Temperature °C	pH	EC (mS/cm)	TDS (mg/l)	Dissolved Oxygen (mg/l)
Hot spring 1	02° 00′ 3.7″	36° 14′ 32″	603	45.1	9.8	0.03	1	12.4
Hot spring 2	01° 43′ 28″	36° 16′ 21″	611	83.6	9.4	1	1	0.04
Hot spring 3	01° 43′ 56″	36° 17′ 11″	616	81	9.2	1	1	0.71

### Measurements of physicochemical parameters

Geographical position of each site in terms of latitude, longitude and elevation was taken using Global Positioning System (GARMIN eTrex 20). The pH for each sampling point was measured with a portable pH-meter (Oakton pH 110, Eutech Instruments Pty. Ltd) and confirmed with indicator strips (Merck, range 5–10). Temperature, Electrical Conductivity (EC), Total dissolved solids (TDS) and dissolved oxygen (DO) were measured on site using Electrical Chemical Analyzer (Jenway - 3405) during sampling. *In situ* temperature was recorded once for each study site and assigned to all the three sample types for that site.

### Sample collection

All samples were collected randomly in triplicates from each hot spring. Water samples were collected using sterile 500 ml plastic containers that had been cleaned with 20 % sodium hypochlorite and UV-sterilized for one hour. Wet sediments were collected by scooping with sterilized hand shovel into sterile 50 ml Falcon tubes. The upper 5 mm from each microbial mat developing on the hot spring water margins was collected into sterile 500 ml plastic jam jars. All samples were preserved on dry ice immediately after sampling, and transported to the laboratory in Jomo Kenyatta University of Agriculture and Technology. Water for nucleic acid extraction (500 ml) was filtered through a 0.22 μM filter membrane (Whatman) and all filter papers containing samples were stored at −80 °C. Pellets were obtained from water samples by re-suspending the filter papers in phosphate buffer solution, and centrifuging 5 ml of the suspension at 13,000 rpm for 10 min. These were used for nucleic acid extraction.

### Nucleic acid extraction

Total community DNA was extracted from all the samples in triplicates; pellets from water samples, 0.2 g of sediment samples and 0.4 g of microbial mat samples, as described by as described by Sambrook et al. [18]. Total RNA was extracted from 0.25 g of sediment and mat samples, and pellets obtained from the water samples (described above), in triplicates using Trizol RNA extraction protocol [19]. The respective nucleic acids extracted from triplicate samples were pooled during the precipitation stage, the pellets were air dried and stored at −20 °C. The pellets were lyophilized to protect them from degradation.

### Synthesis of cDNA from 16S rRNA

cDNA synthesis, amplification and sequencing were performed at Molecular Research DNA Lab (www.mrdnalab.com, Shallowater, TX, USA). The quality of total RNA was assessed using gel electrophoresis. The extracted RNA was dissolved in RNase-free water and subsequently treated to remove DNA contaminants using the Amplification Grade DNase I Kit (Sigma, MO) according to manufacturer’s instructions. cDNA first-strand and second-strand synthesis was done using the Superscript III First-Strand Synthesis SuperMix (Invitrogen, CA) and the Second-strand cDNA Synthesis Kit (BeyoTime, Jiangsu, China), respectively, following manufacturer’s instructions. Single-strand reverse transcription was done to provide template for amplicon libraries using Superscript III (Invitrogen) according to the manufacturer’s protocol, random hexamer primed and with subsequent RNAse H digestion. The Double stranded cDNA synthesis was carried out as described by as described by Urich et al. [20].

### Amplicon library preparation and sequencing

PCR amplification of the 16S rRNA gene V4 variable region was carried out from extracted DNA and cDNA generated from rRNA, using bacteria/archaeal primers 515 F (GTGCCAGCMGCCGCGGTAA) that had barcode and 806R (GGACTACHVGGGTWTCTAAT) according to Caporaso et al. [21]. Amplification proceeded in a 30 cycle PCR using the HotStarTaq Plus Master Mix Kit (Qiagen, USA) with initial denaturation heating at 94 °C for 3 min, followed by 28 cycles of denaturation at 94 °C for 30 s, annealing at 53 °C for 40 s and extension at 72 °C for 1 min, and a final elongation at 72 °C for 5 min. The quality of PCR products was assessed on 2 % agarose gel to determine the success of amplification and the relative intensity of bands. Multiple samples, tagged with different barcodes, were pooled in equimolar ratios based on their DNA concentrations from the gel images. Pooled samples were purified using calibrated Ampure XP beads (Beckman Coulter) for use in library preparation. The pooled and purified PCR products were used to prepare 16S rDNA and cDNA library by following Illumina TruSeq DNA library preparation protocol [22]. Sequencing was performed at MR DNA (www.mrdnalab.com, Shallowater, TX, USA) on a MiSeq 2x300bp Version 3 following the manufacturer’s guidelines.

### Sequence analysis, taxonomic classification and data submission

Sequences obtained from the Illumina sequencing platform were depleted of barcodes and primers using a proprietary pipeline (www.mrdnalab.com, MR DNA, Shallowater, TX) developed at the service provider’s laboratory. Low quality sequences were identified by denoising and filtered out of the dataset [23]. Sequences which were <200 base pairs after phred20- based quality trimming, sequences with ambiguous base calls, and those with homopolymer runs exceeding 6 bp were removed. Sequences were analyzed by a script optimized for high-throughput data to identify potential chimeras in the sequence files, and all definite chimeras were depleted as described previously [24]. All this data filtering was done by the service provider using their pipeline. *De novo* OTU clustering was done with standard UCLUST method using the default settings as implemented in QIIME pipeline Version 1.8.0 at 97 % similarity level. Taxonomy was assigned to each OTU using BLASTn against SILVA SSU Reference 119 database at default e-value threshold of 0.001 in QIIME [25, 26]. Obtained sequences were submitted to the NCBI Sequence Read Archive with SRP# Study accessions: SRP061805. These included SRX1124606: RNA-Seq of Prokaryotes: Alkaline Hot springs and SRX1124607: DNA-Seq of Prokaryotes: Alkaline Hot springs (Additional file 1: Table S1, Additional file 2: Table S2, Additional file 3: Table S3 and Additional file 4: Table S4).

### Statistical analysis

Diversity indices (Shannon, Simpson and Evenness) for each sample were calculated using vegan package version 1.16-32 in R software version 3.1.3 [27]. Community and Environmental distances were compared using Analysis of similarity (ANOSIM) test, based upon Bray-Curtis distance measurements with 999 permutations. Significance was determined at 95 % confidence interval (*p* = 0.05). Calculation of Bray-Curtis dissimilarities between datasets, hierarchical clustering, Non Metric Dimensional Scaling (NMDS), redundancy analysis (RDA) and parameter correlation were carried out using the R programming language [27] and the Vegan package [28]. To support OTU-based analysis, taxonomic groups were derived from the number of reads assigned to each taxon at all ranks from domain to genus using the taxa_summary.txt output from QIIME pipeline Version 1.8.0.

## Results and discussion

### Sampling

Three hot springs of Lake Magadi and Little Magadi were selected based on different temperature and pH levels. Temperatures ranged from 45.1 to 83.6 °C while pH ranged from 9.2 to 9.8. The TDS was above the measurement range for the Electrical Chemical Analyzer; hence all the readings appeared as one (1). The metadata collected before sampling is summarized in Table 1.

### Composition and diversity of the microbial communities

**Fig. 1:**
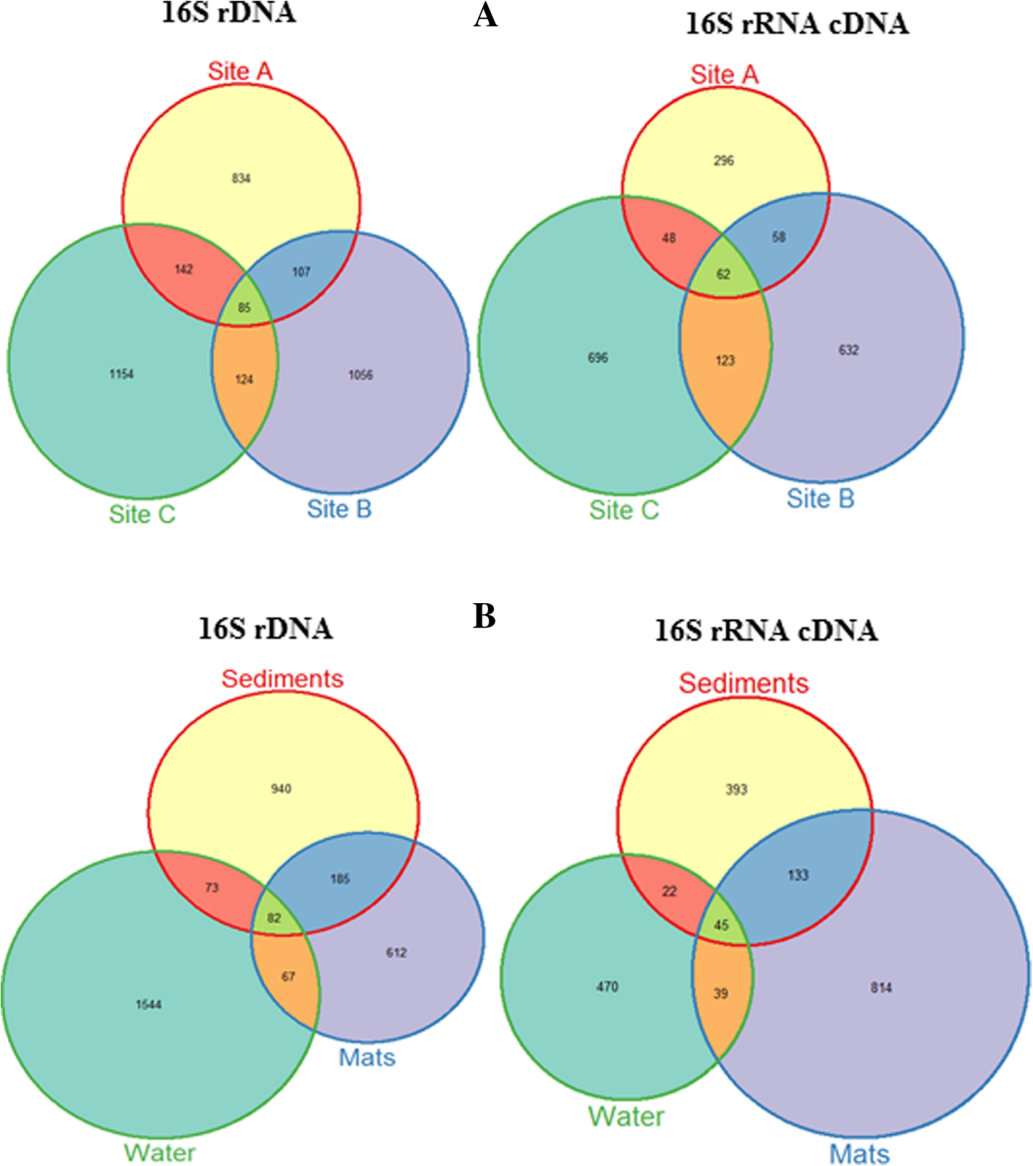
**a** and **b** Venn diagrams showing the distribution of unique and shared OTUs within various sample types in the three sampling sites. The number of OTUs in each hot spring is indicated in the respective *circle*. Site A, B and C represent hot springs 83.6 °C, 81 °C and 45.1 °C respectively

After denoising and demultiplexing, a total of 271,345 and 214,663 sequence reads were generated from 16S rDNA and 16S rRNA cDNA data respectively. Total OTU richness at 3 % distance amounted to 3502 and 1915 OTUs respectively. 85 and 62 OTUs were shared across all hot springs while 82 and 45 OTUs were shared across all sample types in the two data sets respectively. Figure 1a and b shows the distribution of OTUs across hot springs and sample types. For 16S rDNA data, hot spring 83.6 °C and 45.1 °C showed a relatively larger overlap (142 OTUs) than other hot springs, while 45.1 °C harbored most OTUs unique to one hot spring in both datasets.

### Bacterial taxonomic composition analysis

**Fig. 2:**
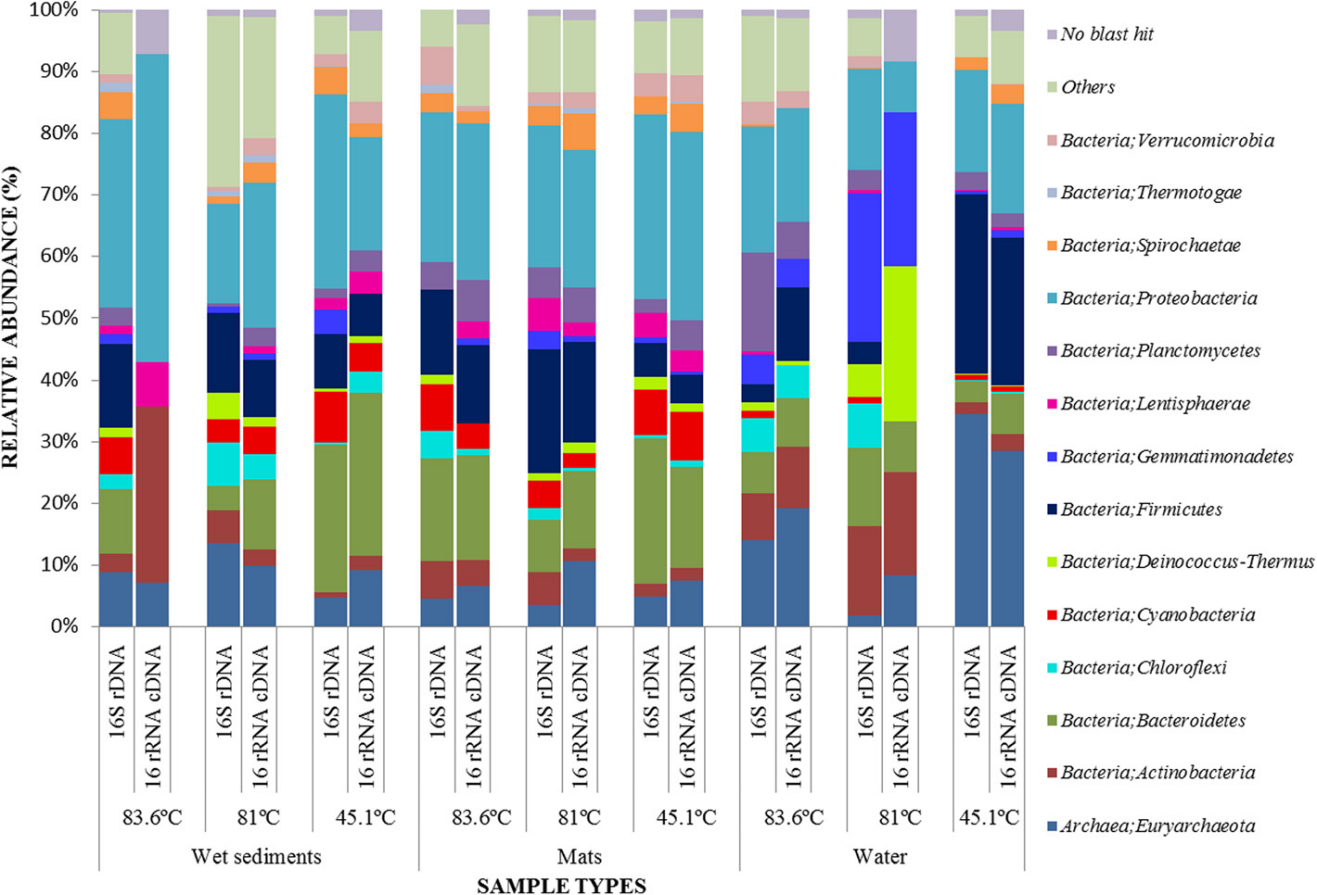
Relative abundance of the most predominant phyla in various samples collected from the hot springs of L. Magadi and Little Magadi. ‘Others’ represent all taxa that scored a relative abundance of below 1 % across all samples in both data sets

16S rDNA OTUs comprised of Bacteria (85.55 %) and Archaea (11.27 %) while 16S rRNA cDNA OTUs comprised of Bacteria (83.22 %) and Archaea (13.95 %). These results suggest that bacteria are the most dominant taxa in L. Magadi and Little Magadi hot springs. The groups with highest relative abundances at phylum level belonged to members of *Proteobacteria* (16–31.4 %), *Firmicutes* (2.8–28.9 %), *Bacteroidetes* (3.4–23.8 %), *Actinobacteria* (1.0–14.4 %), *Cyanobacteria* (1.1–8.3 %, *Chloroflexi* (0.3–7.2 %), and *Deinococcus-thermus* (0.3–5.3 %) (Fig. 2). Other groups scoring high relative abundance in some samples include *Gemmatimonadetes* (24.1 %) in water samples at 81 °C*, Planctomycetes* (15.9 %) in water samples at 83.6 °C, *Lentisphaerae* 5.3 % in mat samples at 81 °C*, Spirochaetae* 4.5 % in wet sediment samples at 45.1 °C*, Thermotogae* (1.7 %) in wet sediment samples at 83.6 °C and *Verumicrobia* (3.9 %) in mat samples at 45.1 °C (Additional file 5: Table S5)*.*

At family level, OTUs were distributed in 292 bacterial families with the most abundant belonging to *Rhodobacteraceae* that scored up to 9.43 % relative abundance in wet sediment samples at 45.1 °C, *Cyanobacteria* Family I (5.89 %), *Desulfonatronaceae* (4.55 %), *Thermaceae* (4.01 %), *Ectothiorhodospiraceae* (3.71 %), *Spirochaetaceae* (3.34 %), *Nitriliruptoraceae* (3.19 %), *Anaerolineaceae* (3.03 %), *Peptococcaceae* (3.03 %) and *uncultured gamma proteobacterium* (3.03 %) in various samples as shown in Additional file 6: Table S6.

Differences in relative abundance were seen as a function of sample type and temperature, with wet sediments harboring the highest taxa. The dominant taxa corresponded with those reported in previous studies conducted on deep sea and marine sediments community composition [29–31]. For example, a review by Brown et al**.** [30] on microbial life in extreme environments that compared metagenome analyses of different high thermal habitats, observed that microbes adapted to these habitats, are different with respect to species abundance and community structure. However, some bacterial taxa such as *Thermotoga, Deinococcus-Thermus* and *Proteobacteria*, were common within the samples under review [30]. These bacterial taxa were also found within the samples from the hot springs of Lakes Magadi and Little Magadi. Species diversity in high temperature environments has been shown to be relatively low [32, 33]. The deep-sea hydrothermal vent chimneys have been found to harbor *Proteobacteria* [34, 35], *Bacteroidetes* and *Planctomycetes* [36].

### Archaeal taxonomic composition analysis

**Fig. 3:**
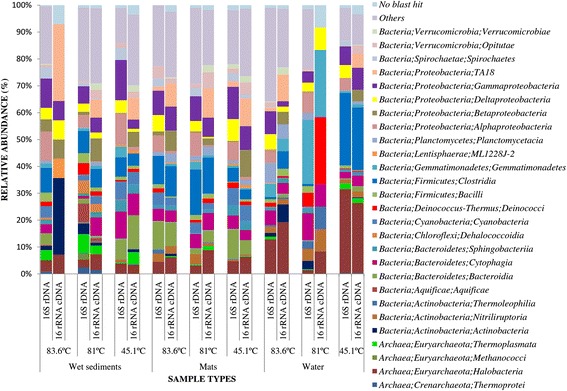
Relative abundance of the most abundant prokaryotic taxa at order level in samples from the hot springs of L. Magadi and Little Magadi. ‘Others’ represent all taxa that scored a relative abundance of below 1 % across all samples in both data sets

The OTUs were distributed among three Archaeal phyla; *Euryarchaeota* (1.76–34.48 %) across all samples*, Crenarchaeota* (up to 2.46 %) and *Thaumarchaeota* (up to 2.78 %) in wet sediment samples at 81 °C (Figs. 2 and 3). At the family level, OTUs were distributed in 24 families with the most abundant belonging to *Halobacteriaceae* (27.06 %) in water samples at 81 °C, *Deep Sea Hydrothermal Vent Gp 6* (*DHVEG-6*) (0.35–5.15 %) across all samples, *South African Goldmine Gp* (*SAGMEG*); *Uncultured Archaeon (*4.01 %*)* and *Methanocaldococcaceae* (2.47 %) in wet sediment samples at 81 °C (Additional file 6: Table S6). *Crenarchaeota* phyla members identified belonged to the families *Desulfurococcaceae* (0.93 %), *Thermoproteaceae* (0.93 %), *Thermofilaceae* (0.61 %) in wet sediment samples at 81 °C and *Sulfolobaceae* (0.42 %) in wet sediment samples at 83.6 °C*,* while *Thaumarchaeota* were mainly assigned to *uncultured archaeon* with up to 1.68 % relative abundance in wet sediment samples at 83.6 °C (Additional file 6: Table S6). Previous studies on thermal groundwater in a thermal field in Russia showed that Archaea is dominated by a novel division in the phylum *Euryarchaeota* related to the order *Thermoplasmatales* (39 % of all archaea) and by another abundant group (33 % of all archaea) related to the phylum *Crenarchaeota.* Both groups are widely spread in hot springs all over the world [37]. Some Archaeal taxa such as *Methanococcus, Thermoprotei* and *Thermococcus* were also common within the samples under review by Brown et al**.** [30]. These are similar to the classes obtained in this study, indicating that Archaea are well adapted to extreme conditions and could be responsible for various functional processes within the ecosystem.

### Active diversity taxonomic composition based 16S rRNA cDNA analysis

#### Bacterial taxonomic composition

The 1913 OTUs of 16S rRNA cDNA data set were dominated by bacterial phyla comprising *Proteobacteria* (8.3–50 %) and *Actinobacteria* (2.0–28.6 %) across all samples*.* The data confirm the predominance of previously defined groups belonging to *Proteobacteria* [38]. Other groups scoring high relative abundance in some samples include *Bacteroidetes* (26.4 % in wet sediment samples at 45.1 °C), *Chloroflexi* (5.3 % in water samples at 45.1 °C), *Firmicutes* (23.9 % in mat samples at 45.1 °C), *Cyanobacteria* (8 % in mat samples at 45.1 °C)*, Gemmatimonadetes* (25 % in water samples at 81 °C)*, Lentisphaerae* (7.1 % in wet sediment samples at 83.6 °C) and *Planctomycetes* (6.6 % in mat samples at 83.6 °C) (Fig. 2).

Unlike 16S rDNA dataset, the most abundant bacterial families found within cDNA dataset belonged to *Methylophilaceae;* scoring up to 8.3 % relative abundance in some samples, *Corynebacteriaceae*, *Dermatophilaceae*, *Micrococcaceae, Nocardioidaceae, Bacteroidaceae; ML635J-40 aquatic group, Sphingomonadaceae*, *Burkholderiaceae*, and *Myxococcales; 0319-6G20, Alcanivoracaceae, Pseudomonadaceae* (7.14 %), *Desulfuromonadales; GR-WP33-58 (5.11 %), Cyanobacteria*; Subsection III; Family I, *Syntrophomonadaceae* (*4.7 %*), *Spirochaetaceae* (3.7 %), *Anaerolineaceae* (3.44 %), *Gemmatimonadetes*; *BD2-11* terrestrial group; uncultured bacterium 3.3 %, *Rhodospirillaceae* (3.3 %) and *Lentisphaerae* (*ML1228J-2; uncultured bacterium*) (*2.6 %*) (Additional file 7: Table S7).

### Archaeal taxonomic composition

16S rRNA cDNA OTUs were distributed among three Archaeal phyla, similar to those obtained from the 16S rDNA dataset. These were *Euryarchaeota* (6.6–28.4 %) across all samples*, Crenarchaeota* (up to 1.38 %) in water samples at 81 °C and *Thaumarchaeota* (up to 1.15 %) in wet sediment samples at 45.1 °C (Fig. 3). The most abundant families belonged to *Halobacteriaceae* (21.31 %), *Deep Sea Hydrothermal Vent Gp 6* (DHVEG-6) (8.59 %), and *Marine Benthic Group D* and *DHVEG-1* (2.29 %). *Crenarchaeota* phyla members identified belonged to the families *Desulfurococcaceae* (1.11 %) in wet sediment samples at 81 °C and *Sulfolobaceae* (0.28 %) in wet water samples at 45.1 °C*,* while *Thaumarchaeota* were mainly assigned to *uncultured archaeon* with up to 1.15 % relative abundance in wet sediment samples at 45.1 °C (Additional file 7: Table S7). Previously, several 16S rRNA gene sequences related to novel Archaea (Euryarchaeota) were retrieved from the alkaline saltern at Lake Magadi [13]. Haloalkaliphilic Archaea related to *Natronomonas*, *Natrialba*, *Natronolimnobius* and *Halorubrum spp*. have also been isolated from Lake Magadi and Inner Mongolian soda lakes [39].

### Microbial richness and diversity indices

Using rarefaction, the same number of sequences from each sample was used in comparison of community alpha and beta diversity measures. Paired t-tests at class taxonomic level of both 16S rDNA and 16 rRNA cDNA indicated that significant differences between samples based on alpha diversity indices whose values obtained were as follows: Shannon diversity index (H’); wet sediment 83.6 °C (7.9 vs 3.8), water 81 °C (9.1 vs 3.6) and Simpson (1/D); wet sediment 83.6 °C (34.54 vs 12.2); water 81 °C (7.76 vs 6) and water 45.1 °C (28.53 vs 10.3) respectively (Table 2).

**Table 2:** Diversity indices computed on all OTU-based microbial taxonomic units within 16S rDNA and 16S rRNA cDNA datasets

Sample ID	Dataset	No. of sequences	No. of OTUs	Shannon (H’)	Simpson (1/D)	Evenness
Wet sediment (83.6 °C)	16S rDNA	38753	238	5.5	34.54	0.658
	16S rRNA cDNA	1625	14	2.6	12.2	0.975
Mats (83.6 °C)	16S rDNA	7575	66	4.2	27.41	0.475
	16S rRNA cDNA	21451	212	5.3	32	0.632
Water (83.6 °C)	16S rDNA	19186	680	6.5	14.88	0.356
	16S rRNA cDNA	5124	151	5.0	18.1	0.594
Wet sediment (81 °C)	16S rDNA	36047	324	5.8	22.57	0.427
	16S rRNA cDNA	67235	361	5.9	53.6	0.636
Mats (81 °C)	16S rDNA	24252	282	5.6	34.55	0.61
	16S rRNA cDNA	33751	349	5.8	34.3	0.538
Water (81 °C)	16S rDNA	36126	568	6.3	7.76	0.278
	16S rRNA cDNA	638	12	2.5	6	0.866
Wet sediment (45.1 °C)	16S rDNA	52115	509	6.2	35.09	0.577
	16S rRNA cDNA	11471	87	4.4	27.7	0.784
Mats (45.1 °C)	16S rDNA	37446	382	6.0	22.45	0.785
	16S rRNA cDNA	48578	375	6.0	37.4	0.572
Water (45.1 °C)	16S rDNA	19789	377	6.0	28.53	0.461
	16S rRNA cDNA	24790	352	5.9	10.3	0.346

Total microbial diversity based on 16S rDNA and 16S rRNA cDNA ANOSIM at order level showed that there were significant differences in microbial community structure in the samples at 95 % level of confidence (*P* value, 0.009), and 0.383 R statistic value while active microbial diversity based on 16S rRNA cDNA had (*P* value, 0.01), and 0.333 R statistic value. This could be attributed to differences in temperature and pH of the specific sites during sampling. Samples from 45.1 °C harbored more closely related populations because it had less extreme conditions as compared to the two other hot springs.

Distance based redundancy analysis showed that the microbial community evenness significantly differed from each site for both 16S rDNA and 16S rRNA cDNA. 16S rDNA ordination of the three sample types showed a significance of 0.017 and while 16S rRNA cDNA dataset showed a significance of 0. 011. Samples from each site clustered close to each other in separate quadrants indicating high beta diversity between the three sampling sites.

**Fig. 4:**
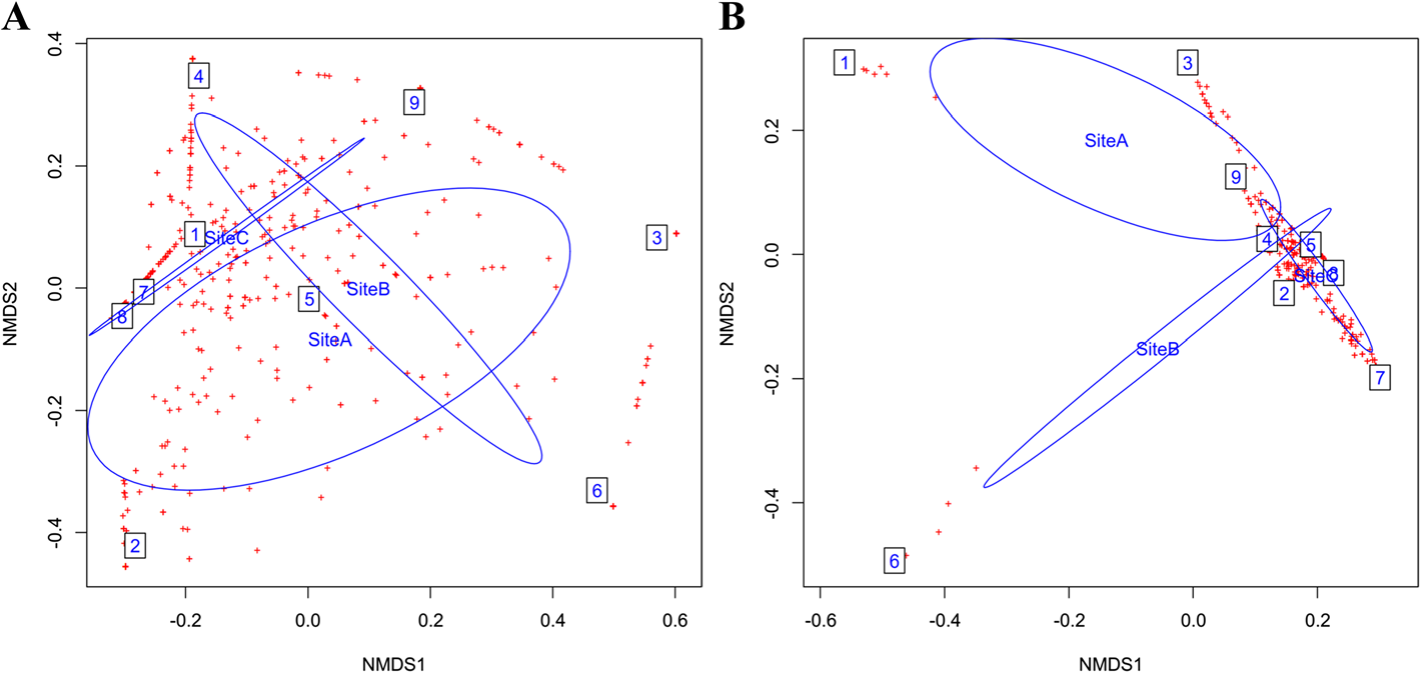
**a** Non-metric multidimensional scaling (NMDS) based on Bray-Curtis dissimilarities between microbial compositions of 16S rDNAdataset, grouped according to sampling sites. Site A, B and C represent hot springs 83.6 ºC, 81 ºC and 45.1 ºC respectively. The boxes 1 - 3 represent wet sediments, mats and water samples from 83.6 ºC, 4 - 6 represent wet sediments, mats and water samples from 81 ºC and 7 - 9 represent wet sediments, mats and water samples from 45.1 ºC. **b** Non-metric multidimensional scaling (NMDS) based on Bray-Curtis dissimilarities between microbial compositions of 16S rRNA cDNAdatasets grouped according to sampling sites. Site A, B and C represent hot springs 83.6 ºC, 81 ºC and 45.1 ºC respectively. The boxes 1 - 3 represent wet sediments, mats and water samples from 83.6 ºC, 4 - 6 represent wet sediments, mats and water samples from 81 ºC and 7 - 9 represent wet sediments, mats and water samples from 45.1 ºC

**Fig. 5:**
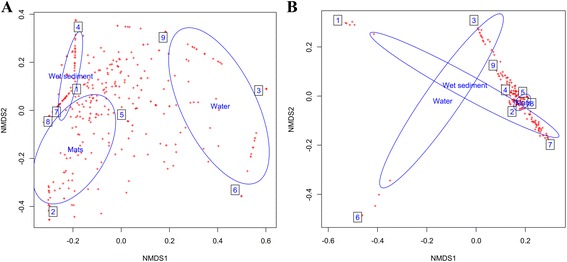
**a** Non-metric multidimensional scaling (NMDS) based on Bray-Curtis dissimilarities between microbial compositions of 16S rDNAdatasetgrouped according to sample types. Each ellipse represents a set of the three sample types collected from each hot spring. The boxes 1 - 3 represent wet sediments, mats and water samples from 83.6 ºC, 4 - 6 represent wet sediments, mats and water samples from 81 ºC and 7 - 9 represent wet sediments, mats and water samples from 45.1 ºC. **b** Non-metric multidimensional scaling (NMDS) based on Bray-Curtis dissimilarities between microbial compositions of 16S rRNA cDNA dataset grouped according to sample types. Each ellipse represents a set of the three sample types collected from each hot spring. The boxes 1 - 3 represent wet sediments, mats and water samples from 83.6 ºC, 4 - 6 represent wet sediments, mats and water samples from 81 ºC and 7 - 9 represent wet sediments, mats and water samples from 45.1 ºC

NMDS analyses supported by OTU and taxonomic composition, divide the datasets into three ellipses: one for each hot spring. Some microbial taxa were shared between habitats in both 16S rDNA and 16S rRNA cDNA derived datasets. This scenario was more pronounced in 16S rDNA - derived dataset, indicating that DNA pool contained a “seed bank” of inactive and sporulating organisms [40], while fewer taxa were active within the ecosystem as shown in the 16S rRNA cDNA derived dataset (Fig. 4). Similar results were observed in a study on Ethiopian soda lakes where NMDS analyses supported both by OTU and taxonomic composition; divided the datasets into six well-separated habitats with relatively few OTUs that were shared between more than one or two habitats [41]. The taxa were also observed to cluster according to sample types (i.e. wet sediments, microbial mats and water samples). There was an overlap of taxonomic clusters between wet sediments and microbial mats from the three sample types in both 16S rDNA and 16S rRNA cDNA derived datasets**.** However, water samples formed separate clusters from the other two sample types in both 16S rDNA and 16S rRNA cDNA datasets (Figs. 4 and 5).

**Fig. 6:**
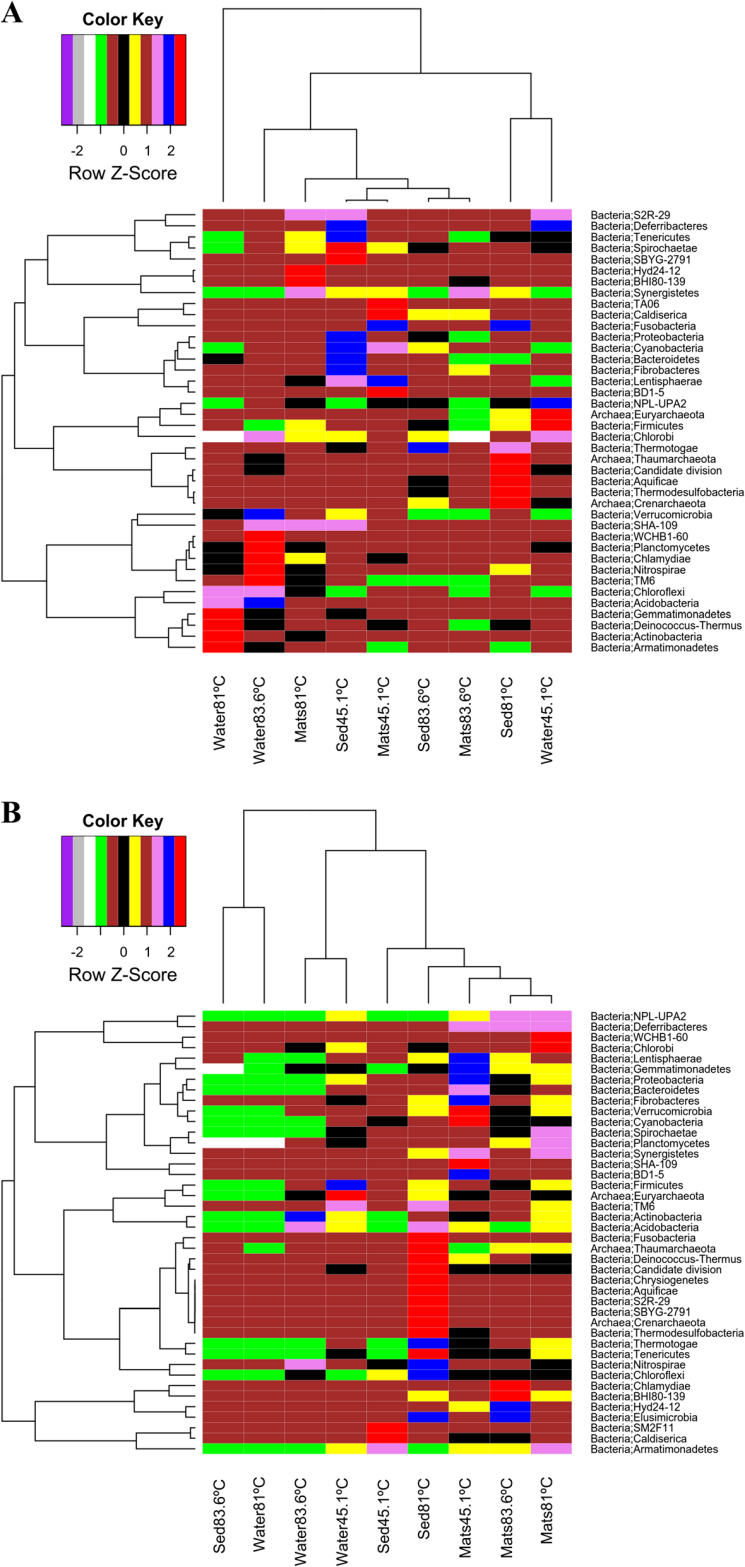
**a** Hierarchical clustering of 16S rDNA samples collected from the three hot springs of L. Magadi and Little Magadi. Phylum level was chosen to be used in hierarchical clustering to assess the relationships between samples and taxa. “Sed” represent wet sediment samples from respective temperature. **b** Hierarchical clustering of 16S rRNA cDNA samples collected from the three hot springs of L. Magadi and Little Magadi. Phylum level was chosen to be used in hierarchical clustering to assess the relationships between samples and taxa. “Sed” represent wet sediment samples from respective temperature

Hierarchical clustering between samples collected from lake Magadi and Little Magadi revealed samples from the two hot springs in Little Magadi “*Nasikie eng’ida”* were closer than samples from the hot spring in the main lake (Fig. 6 (a and b) and (Additional file 8: Figure S1 and Additional file 9: Figure S2).

## Conclusion

The combined findings of this study, show that estimated diversity and richness within the hot spring samples were found to be as high as those found in other environments such as soil and deep-sea hydrothermal environments [42]. The results confirm that different groups of microorganisms have the capacity to adapt and thrive even in the most hostile environments. Some of these groups (*Acidobacteria; Blastocatella, Bryobacter* and *Telmatobacter* genera*, Bacteroidetes; Bacteroidales, Rhodothermaceae, Flavobacteriaceae, Sphingobacteriales* and *Chloroflexi; Dehalococcoidales and Thermomicrobia*) also obtained from previous similar studies were reported to have a fermentative ability [41]. It was observed that from the cDNA dataset, photosynthetic taxa were represented by Cyanobacterial genera *Leptolyngbya* and *Lyngbya*, among other uncultured groups. Primary production within the hot springs is probably supported by some groups of non-sulfur purple bacteria from the family *Rhodobacteraceae* (specifically the genera *Roseobacter* that scored 1.1 % relative abundance), and purple sulfur bacteria from the family *Ectothiorhodospiraceae* present across samples at different relative abundance. The presence of *Planctomycetes* within samples could be an indicator that anaerobic ammonium oxidation may be another metabolic pathway supporting primary production in the low-oxygen, saline environment, since the dissolved oxygen concentration of the sampling sites ranged between 0.04 and 12.4 mg/l. *Actinobacteria* and *Firmicutes* are believed to have adaptive advantage under low-nutrient conditions of the highly alkaline, saline hot springs hence their high relative abundance levels. The presence of sulfate reducers in the family *Desulfohalobiaceae* (mainly *Desulfonatronovibrio*), suggested an internal sulfur cycle within the lake, as previously suggested for Khadin, Tuva, Russia and Natron soda lakes [42, 43]. Taxa typical for highly specialized metabolisms that were encountered in this study include *Nitriliiruptor*, known for their ability to catabolize nitriles or cyanides [44] and heterotrophic *Oceanospirillaceae* (*Marinospirillum*). Other functional taxa encountered include aerobic heterotrophs (e.g. *Bacteroidetes*, *Marinicella*) and fermentative anaerobes such as *Thermoplasmatales* among other uncultured groups. *Euryarchaeota* members were clustered into the classes, *Halobacteria*, *Methanobacteria*, *Methanomicrobia*, *Methanococci*, *Thermococci* and *Thermoplasmata* while *Crenarchaeota* phyla comprised *Thermoprotei* class. Previously, *Methanobacteria* and *Methanomicrobia* have been reported in oilfields, while *Halobacteria* and *Thermoprotei* have been reported in petroleum reservoirs [45]. Some *Halobacteria* members are important in organic fertilizer production industry as Lignin decomposers [46]. However, most of the genera identified in this study are known to be heterotrophs responsible for the primary degradation of organic matter [39]. The actual function of microbial taxa reported in this study could further be explored and established using culture dependent methods as well as mRNA transcripts.

In conclusion, this study presented microbial diversity analysis of samples collected from the hot springs of L. Magadi and Little Magadi based on both DNA and RNA, using Illumina Sequencing Technology. The results showed comparable profiles of microbial community using 16S rDNA and 16S rRNA cDNA derived datasets, hence indicating that the observed diversity is real. The findings showed a broad microbial distribution with water from the spring at 83.6 °C found to be the richest sample, constituting 680 observed species. Despite the fact that the sampling environment is multi-extreme due to high pH, temperature, and salinity, this study shows that there are stable and active microbial communities that have adapted to this environment. Culture dependent studies in future will help us unravel the survival mechanisms used by these polyextremophiles.

## Additional files

Additional file 1: Table S1. Total diversity environmental data sheet of the samples collected from the hot springs of L. Magadi and Little Magadi (XLS 25 kb) Additional file 2: Table S2. Active diversity environmental data sheet of the samples collected from the hot springs of L. Magadi and Little Magadi (XLS 24 kb) Additional file 3: Table S3. Total diversity Sequence Read Archive metadata sheet of the samples collected from the hot springs of L. Magadi and Little Magadi (XLSX 50 kb) Additional file 4: Table S4. Active diversity Sequence Read Archive metadata sheet of the samples collected from the hot springs of L. Magadi and Little Magadi. (XLSX 50 kb) Additional file 5: Table S5. Relative abundance of microbial taxa at phylum level in various samples collected from the three hot springs (83.6, 81 and 45.1 °C) of L. Magadi and Little Magadi. ‘No blast hit/Others’ represent unclassified sequences in both data sets. (XLSX 17 kb) Additional file 6: Table S6. Total diversity relative abundance of microbial taxa at family level in various samples collected from the three hot springs (83.6, 81 and 45.1 °C) of L. Magadi and Little Magadi. ‘No blast hit/Others’ represent unclassified sequences in both data sets. (XLSX 45 kb) Additional file 7: Table S7. Active diversity relative abundance of microbial taxa at family level in various samples collected from the three hot springs (83.6, 81 and 45.1 °C) of L. Magadi and Little Magadi. ‘No blast hit/Others’ represent unclassified sequences in both data sets. (XLSX 32 kb) Additional file 8: Figure S1. Comparative analysis (UPGMA similarity tree) of total microbial diversity of various sample types within hot springs of L. Magadi and Little Magadi. (DOCX 55 kb) Additional file 9: Figure S2. Comparative analysis (UPGMA similarity tree) of active microbial diversity of various sample types within hot springs of L. Magadi and Little Magadi. (DOCX 56 kb)
