# Supplementary material for: Bacteria and Archaea diversity within the hot springs of Lake Magadi and Little Magadi in Kenya
Source: BMC Microbiol. 2016 Jul 7;16:136. doi: 10.1186/s12866-016-0748-x (PMC4936230; doi:10.1186/s12866-016-0748-x)
Supplement: Additional file 8: Figure S1. — Comparative analysis (UPGMA similarity tree) of total microbial diversity of various sample types within hot springs of L. Magadi and Little Magadi. (DOCX 55 kb) [file 12866_2016_748_MOESM8_ESM.docx]

Mats (45.1ºC)

Sediments (45.1ºC)

Sediments (83.6ºC)

Sediments (81ºC)

Mats (81ºC)

Mats (83.6ºC)

Water (45.1ºC)

Water (81ºC)

Water (83.6ºC)

**DNA data UPGMA Tree**

0.3696

0.3983

0.3602

0.3937

0.4113

0.3823

0.4308

0.4157
