# Supplementary material for: Bacteria and Archaea diversity within the hot springs of Lake Magadi and Little Magadi in Kenya
Source: BMC Microbiol. 2016 Jul 7;16:136. doi: 10.1186/s12866-016-0748-x (PMC4936230; doi:10.1186/s12866-016-0748-x)
Supplement: Additional file 9: Figure S2. — Comparative analysis (UPGMA similarity tree) of active microbial diversity of various sample types within hot springs of L. Magadi and Little Magadi. (DOCX 56 kb) [file 12866_2016_748_MOESM9_ESM.docx]

Mats (81ºC)

Mats (45.1ºC)

Sediments (81ºC)

Mats (83.6ºC)

Sediments (45.1ºC)

Water (83.6ºC)

Water (45.1ºC)

Water (81ºC)

Sediments (83.6ºC)

0.3914

0.3763

0.385

0.4104

0.4249

0.3956

0.4592

0.3999

**cDNA data UPGMA Tree**
